# Influence of COVID‐19 pandemic on research and fieldwork: Perspectives from Ghana

**DOI:** 10.1002/hsr2.906

**Published:** 2022-10-25

**Authors:** Fatima Amponsah Fordjour

**Affiliations:** ^1^ Department of Microbiology University for Development Studies (UDS) Tamale Ghana; ^2^ College of Science Kwame Nkrumah University of Science and Technology (KNUST) Kumasi Ghana

**Keywords:** COVID‐19, fieldwork, pandemic, research

## Abstract

The insurgence of COVID‐19 has received all attention at the detriment of research on most diseases. Procedures and protocols devised to curb the pandemic continues to affect lives and work. As most countries prepare to tune back to normalcy, working conditions undoubtedly will not be the same as the World Health Organization still urges nations to scale up procedures and strategies against the pandemic. As research groups and projects across the globe and especially in Ghana begins work, these protocols must be fashioned into their study protocol before approval will be granted by ethical institutions. This has led to increase in research cost, with additional responsibilities aside their normal research activities. This perspective clearly points out the impact of the pandemic on research, especially fieldwork.

## 1 INTRODUCTION AND THE EFFECT OF THE PANDEMIC ON NEGLECTED TROPICAL DISEASES (NTDs)

In this era of global chaos because of the ever‐changing virus (corona virus), the world has entered a stage where all forms of activities must adopt and adapt to conditions that will make working possible. Corona virus disease (COVID‐19) is an infectious disease caused by severe acute respiratory syndrome coronavirus 2 (SARS‐CoV‐2).^1^ The virus is mainly spread between people during close contact, most often through small droplets produced by coughing, sneezing, and talking. Before the COVID‐19 pandemic, organizations and all research related activities had planned systems of operations. However, this is not so now, as individuals and organizations must put in place measures to; (1) adhere to COVID‐19 preventive protocols, (2) change methods of operation (mostly from in person to online, or partially virtual) and (3) increase accessibility of their services/products or research to their target population. Amidst all of these, research and specifically scientific research has gone through a lot of revolution in this era, changing the decisions of individuals and organizations about; (1) grant/award size (2) means of reaching target population, (3) eligible applicants for these awards and (4) the timeliness for the awards and deliveries.

The World Health Organization (WHO) has urged all countries to try as much as possible to vaccinate about 70% of their population.^2^

NTDs are a group of preventable and treatable diseases that affect 1.5 billion people, 40% of whom live in Africa.^3^ Irrespective of classifications, the vast majority of NTDs are prevalent in tropical and subtropical regions of Africa, America, Asia, and Oceania.^4^ However, some of them historically extend beyond these borders. The 5 most common NTDs in Ghana include lymphatic filariasis, onchocerciasis, schistosomiasis, trachoma and soil transmitted helminths.^4^ In 1997, the world health assembly met to deliberate on the preventive measures for most of these diseases. From WHO's expected goals, most NTDs were to be eradicated by 2020. However, these goals could not meet target due to many factors with COVID‐19 been a major player. Other factors include mass drug administration coverage, cost, and logistics especially in Sub Saharan Africa. In 2019, the WHO aimed at revamping measures to end most of the NTDs by providing adequate logistics, education and visibility until COVID‐19 became a global concern, halting all other programs and health activities. This has been one of the biggest blows to NTDs prevention. Efforts channeled towards COVID‐19 responses have tampered with the progress made towards NTDs elimination but with targeted and scaled‐up efforts, NTDs could be eradicated by 2030 as targeted by WHO.^5^

## 2 EFFECT OF COVID‐19 ON FIELDWORK

The pre‐eminence of the pandemic was graced with research organizations and units operating by ordered mode of activities regarding meetings and screening. This notwithstanding, during and after the pandemic, all these institutions had to meet based on standard and accepted method for meetings. This made research and active screening extremely difficult or nearly impossible. Nevertheless, because research works could not be entirely halted, individuals and organization developed systematic approach towards meeting and screening individuals for research. At this point, having trained community health volunteers (CHVs) became a gold mine, especially in NTDs as these people live in the endemic communities and are together with the target population. Most research groups relied on the CHVs in their activities for screening. Individuals identified by the CHVs to be eligible participants were first registered, and the list submitted to heads of research groups for further confirmation. This procedure was followed by either virtual interview or time limited in‐person interview with the study team. Qualified participants were then listed for further confirmation. One negative impact of this approach was that it reduced the number of eligible participants eligible for the study and thus research groups had to screen for a long time before getting sample size for their study.

Another impact of the pandemic on research activities is the direction of almost all resources toward the pandemic neglecting other equally important diseases such as malaria, cholera and NTDs. Due to the sudden outbreak, the WHO and other bodies focused on the pandemic putting almost all others on the shelf to be revisited at a later time. This caused an insurgence in malaria and cholera cases which are also seasonal diseases mostly in the rainy seasons especially in Africa.

## 3 RESUMPTION FROM LOCKDOWNS AND INDOOR RESEARCH

After months of lockdown and indoor living, active screening in research must continue. This is very paramount in that, such activities lead to the discovery of new infections, population at risk and helps in setting strategies to combat them. Although, outdoor programs and activities have been allowed in various countries and communities, it is still essential for research teams and organizations to develop protocols on COVID‐19 as an addition to their main study protocol.

It is unarguably true that the pandemic positively became a gold mine for some institutions. In that products and services needed to survive the pandemic faced rocketing prices. Hence making research more expensive with increase in the pandemic virus.

## 4 THE FUTURE WITH COVID‐19

From WHO's bulletin, it is pleasing to see that some countries with the lowest vaccination rates are now making up ground, especially in Africa. Currently some countries still have less than 10% coverage, most of which are facing humanitarian emergencies. However, much more needs to be done, as one‐third of the world's population remains unvaccinated, including two‐thirds of health workers and three‐quarters of older adults in low‐income countries. In January 2022, WHO, UNICEF, and industry partners established the COVID‐19 Vaccine Delivery Partnership to accelerate vaccine coverage in the 34 countries that were below 10% coverage, most of these countries been from Africa. Suggestions from other studies have advised the coming together of African health experts to share ideas on the pattern of the pandemic in Africa and how to overcome challenges that can affect the achievement of the Sustainable Development Goals.^6^, ^7^

## 5 CONCLUSION AND RECOMMENDATION

The COVID‐19 pandemic had significant impact on the fight against NTDs in Ghana, however, it is paramount that already NTDs do not become more sidelined. With the nonabating pandemic, novel ways to resume the NTD programmes ought to be explored after a risk benefit assessment by the government of Ghana. This can be harnessed by channeling information through our media (TV, radio, and others). The need for partnerships and collaborations cannot be overemphasized in reaching the roadmap of ending NTDs by 2030 as postulated by WHO. Just like every other country, revamping the capacity Ghana health system to match up with the task of curtailing the socioeconomic impact of NTDs in the country will be a game changer (Table 1).

**Table 1:** Showing the treatment coverage analysis of NTDs in Ghana based on available data from Master Plan for Neglected Tropical Diseases Programme, Ghana before COVID‐19 (2016)

NTDs	Coverage (%)	Treatment received so far
Lymphatic filariasis (LF)	74	1.32 million people
Onchocerciasis	84	4.33 million
Schistosomiasis	67	2.64 million
Soil transmitted helminths (STHs)	41	4.04 million
Trachoma	100	No treatment has been received currently due to complete eradication

Abbreviation: NTD, neglected tropical diseases.

## AUTHOR CONTRIBUTIONS

**Fatima Amponsah Fordjour**: conceptualization; data curation; supervision; validation; visualization; writing – original draft; writing – review & editing.

## CONFLICT OF INTEREST

The author declares no conflict of interest.

## TRANSPARENCY STATEMENT

The lead author Fatima Amponsah Fordjour affirms that this manuscript is an honest, accurate, and transparent account of the study being reported; that no important aspects of the study have been omitted; and that any discrepancies from the study as planned (and, if relevant, registered) have been explained.

## Data Availability

Data sharing not applicable to this article.
